# Inebriety

**Published:** 1887-06

**Authors:** 


INEBRIETY —Dr. T. D. Crothers, the able editor of the *Quarterly Journal of Inebriety* and chairman of the American Commission kindly sends a circular from which we extract the following:

"The Council of the English Society for the Study and Cure of Inebriety have completed arrangements for an International Medical Congress, to be held at Westminister Hall, London, July 5th and 6th, 1887.

The object of this Congress is to present and discuss the problems of Inebriety Medically, and from a purely *scientific standpoint*, by the best authorities, thus laying the foundation for a broader and more exact study of this subject.

Papers and addresses are promised from a large number of the most distinguished Physicians.

